# The association between wearable activity monitor metrics and performance status in oncology: a systematic review

**DOI:** 10.1007/s00520-021-06234-5

**Published:** 2021-06-12

**Authors:** Milan Kos, Esther N. Pijnappel, Laurien M. Buffart, Britt R. Balvers, Caroline S. Kampshoff, Johanna W. Wilmink, Hanneke W. M. van Laarhoven, Martijn G. H. van Oijen

**Affiliations:** 1 Department of Medical Oncology, Cancer Center Amsterdam, Amsterdam UMC, University of Amsterdam, Meibergdreef 9, Amsterdam, The Netherlands; 2 Department of Physiology, Radboud Institute for Health Sciences, Radboud University Medical Center, Philips van Leydenlaan 15, Nijmegen, The Netherlands; 3 Department of Medical Oncology, Cancer Center Amsterdam, Amsterdam UMC, Vrije Universiteit Amsterdam, De Boelelaan 1117, Amsterdam, The Netherlands; 4 Center for Quality of Life, The Netherlands Cancer Institute, Amsterdam, The Netherlands

**Keywords:** Cancer, Performance status, Physical activity, Physical function, Sedentary behavior, Wearable activity monitor

## Abstract

**Purpose:**

The expanding armamentarium of wearable activity monitors (WAMs) offers new opportunities to supplement physician-assessed performance status (PS) with real-life patient activity data. These data could guide clinical decision making or serve as a measure of treatment outcome. However, information on the association between physical activity (PA) and sedentary behavior (SB) monitored with wearables (i.e., WAM metrics) and PS in patients with cancer is needed. Therefore, we conducted a systematic review to examine the association between WAM metrics and PS in patients with cancer.

**Methods:**

We searched MEDLINE and Embase for studies that assessed the association between WAM metrics and performance status among adults with cancer. We extracted information on study design and population, WAM type and different activity metrics, outcome definitions, and results. Included studies were subjected to risk of bias assessment and subsequent best evidence synthesis.

**Results:**

Fourteen studies were included in this review. All studies reported on different combinations of WAM metrics including: daily steps (*n* = 8), SB (*n* = 5), mean activity counts (*n* = 4), dichotomous circadian rest-activity index (*n* = 3), and time spent in moderate-to-vigorous PA (MVPA) (*n* = 3). Much heterogeneity was observed regarding study population, WAM used, and reporting of results. We found moderate evidence for a positive weak-to-moderate association between WAM-assessed PA and PS and a weak-to-moderate negative association between WAM-assessed SB metrics and PS.

**Conclusion:**

Weak-to-moderate associations between WAM metrics and PS suggest that WAM data and physician-assessed PS cannot be used interchangeably. Instead, WAM data could serve as a dynamic and objective supplement measurement of patients’ physical performance.

**Supplementary Information:**

The online version contains supplementary material available at 10.1007/s00520-021-06234-5.

## Introduction

Patients’ performance status is a significant prognostic and predictive factor for clinically relevant outcomes, such as progression-free and overall survival of patients with cancer [1–3]. It therefore is one of the key inclusion criteria for clinical trials and often serves as stratification factor in trial design and analyses. Moreover, in daily clinical practice it is used to decide whether a patient is fit for systemic therapy [4], or eligible for early phase clinical trials. Patients’ performance status is determined by healthcare professionals using either the Karnofsky Performance Status (KPS) or the Eastern Cooperative Oncology Group Performance Status (ECOG-PS, also known as WHO PS) [5, 6]. Both methods have proven their clinical relevance over the past decades and are widely used. However, these methods also present with potential bias and limitations [7]. First, performance status scoring depends on the oncologists subjective rating of a patient’s health and functioning with no standardized process for this assessment, making it prone to under- and overestimation, and inter-observer differences [8–12]. Second, performance status assessment may be susceptible to response and recall bias as it relies on patient-reported physical activity and functioning [13]. Third, both KPS and ECOG-PS are static measurements that are only captured during scheduled visits, whereas patient’s physical performance is a dynamic process that may change on a daily basis during the course of treatment. As a result, recent reviews have accentuated the need for a tool that can assess patient’s physical performance objectively in a more dynamic fashion [7, 14].

The expanding armamentarium of wearable activity monitors (e.g., accelerometers, pedometers, fitness trackers, smartwatches, and smartphones) offers new opportunities to supplement physician-assessed performance status with objective assessments of physical activity and sedentary behavior, which are passively gathered in a non-obtrusive manner. It is even hypothesized that wearable activity monitor metrics might prove superior to clinician-rated performance status or patient-reported data in terms of accurately discriminating between the heterogeneous spectrum of cancer patients [7, 14]. Therefore, wearable activity monitor-derived data may assist healthcare professionals in making treatment decisions (e.g., mono vs doublet vs triplet chemotherapy) for individual patients [15, 16] and could be useful in assuring that performance status of patients enrolled in clinical trials is recorded accurately [7]. Multiple recent clinical studies have demonstrated the feasibility of using wearable activity monitors to assess physical activity and sedentary behavior in patients with cancer. However, no aggregated evidence is available about the use of wearable activity monitor-derived physical activity and sedentary behavior metrics to supplement physician-assessed performance status. As a first step towards this purpose, we conducted a systematic review on the association between wearable activity monitor physical activity and sedentary behavior metrics and performance status in patients with cancer.

## Methods

A systematic review of available literature was conducted in agreement with the guideline for preferred reporting items for systematic reviews and meta-analyses (PRISMA statement) [17]. This review has been registered in PROSPERO (CRD4202013865).

### Literature search

MEDLINE® and Embase databases were searched from inception until April 2020 to identify all relevant published articles. An experienced clinical librarian from the Amsterdam UMC was consulted for the development of the search strategy. Relevant keywords included terms related to wearable activity monitor metrics AND cancer population AND wearable activity monitors (e.g., fitness trackers, smartwatches, accelerometers, pedometers, actigraphs, and inclinometers). The complete search strategies are presented in Supplementary Table 1. Moreover, cross-referencing was performed to identify additional relevant studies for the systematic review.

### Eligibility criteria

Studies were included if they (1) were conducted among adults (≥ 18 years) with cancer, (2) objectively measured physical activity or sedentary behavior using wearable activity monitors in the outpatient setting, (3) measured physician-assessed performance status, (4) quantitatively assessed an association between wearable activity monitor metrics and clinician-assessed performance status, and (5) had a full text available in English.

### Definitions of wearable activity monitor metrics for physical activity, sedentary behavior, and circadian rest-activity rhythm

Many different activity-related wearable activity monitor metrics are being used in research and reported wearable activity monitor metrics often depend on the used device. Four main categories of wearable activity monitor metrics relevant for this review can be identified: (1) accelerometer-related activity count-based metrics that capture the duration and intensity of accelerations in counts per minute and/or hours. Moreover, these intensities can be used to determine absolute or relative time spent in sedentary behavior, light physical activity (LPA), or moderate-to-vigorous physical activity (MVPA) based on predefined cut-points. (2) Posture-based measures that define hours of percentage of time per day spent sitting/lying (i.e., sedentary), standing, or stepping. (3) Steps-based measurement that estimate the number of steps per day using an algorithm that determines whether accelerometer measurements meet the threshold to be counted as a step. (4) Circadian rhythm rest-activity actigraphy parameters like, the dichotomous in-bed vs out-of-bed index (I < O) and mean activity level (MeanAct).

### Selection process and eligibility criteria

Titles and abstracts of articles identified by the electronic database searches were extracted and duplicates were removed. Two reviewers (MK and EP or BB) independently screened the records of the initial search to select potentially relevant articles that were subsequently subjected to full-text screening for eligibility. Any discrepancies between reviewers were discussed in person. If no agreement was reached, discrepancies were referred to a third reviewer (MvO) before a final decision was made on inclusion.

### Data extraction

Data on study design and population, physical activity/sedentary behavior measurement characteristics and protocols, wearable activity monitors, and outcome measures were extracted using a standardized form including the following items: first author, year of publication, country, study design, number of included patients, type of cancer and disease stage, current treatment, comorbidities, performance status scale used, measure of physical activity or sedentary behavior (including definitions and cutoff points), devices used for physical activity/sedentary behavior measurements, wear location of devices, statistical methods and analyses, and results on association between wearable activity monitor metrics and performance status. If point estimates (e.g., mean, median) were only depicted in figures, authors were first contacted and asked to provide these point estimates. If we did not receive data from the authors, we used open source software WebPlotDigitizer (version 4.3) to estimate the point estimates and corresponding measures for variability (e.g., IQR or SD) from the figures [18–20]. The widely used empirical classifications proposed by Evans were used for interpreting correlation strengths [21]. Correlation coefficients of 0–0.19 were interpreted as very weak, 0.2–0.39 as weak, 0.4–0.59 as moderate, 0.6–0.79 as strong, and 0.8–1 as very strong.

### Risk of bias assessment

The risk of bias of included studies was scored independently by two reviewers (MK and EP) using a risk of bias assessment tool based on the guideline for assessing quality in prognostic studies [22]. These guidelines comprise 6 potential biases (i.e., study participation, study attrition, prognostic factor measurement, outcome measurement, confounding measurement and account, and analysis) and were applied on the basis of relevance to this systematic review. Subsequently, the potential biases were translated into a 15-point quality criteria list, based on previously published risk of bias criteria lists (Supplementary Table 2) [23–26]. Furthermore, quality items were categorized as informative (I, 5 items) or validity/precision (V/P, 10 items) [23, 25]. If the study provided adequate information and met the criterion of the quality item, one single point was appointed. If the study provided insufficient information or did not meet the criterion of the quality item, no point was appointed. If the study referred to another article for relevant information regarding the quality item, that article was reviewed to score the item. Disagreements regarding the risk of bias assessment were resolved by discussion and, if no agreement could be reached, by consulting the third reviewer (MvO). A total risk of bias score was calculated for each included study by dividing the amount of positively scored validity/precision items by the total amount of validity/precision items (i.e., 10), resulting in a score between 0 and 1, presented as a percentage. The five informative items were not included in the final calculation, as these items represent descriptive information only [23, 25]. In line with previous reviews [23, 27], a study with a score ≥ 70% was considered to be of “low risk of bias,” whereas as a study with a score < 70% was considered to be of “high risk of bias” [23, 27].

### Level of evidence

A 3-level scoring system for best evidence synthesis based on the number, methodological quality, and consistency of outcomes of the different studies was applied to synthesize the methodological quality of the included studies [23, 27, 28]: (1) strong evidence; provided by general consistent findings in multiple (≥ 2) studies with low risk of bias, (2) moderate evidence; provided by general consistent findings in 1 study with low risk of bias and 1 or more studies of high risk of bias or general consistent findings in multiple (≥ 2) studies with high risk of bias, and (3) insufficient evidence; only one study available or inconsistent findings in multiple (≥ 2) studies. Results were considered general consistent when at least 75% of studies showed results in the same direction.

## Results

**Fig. 1:**
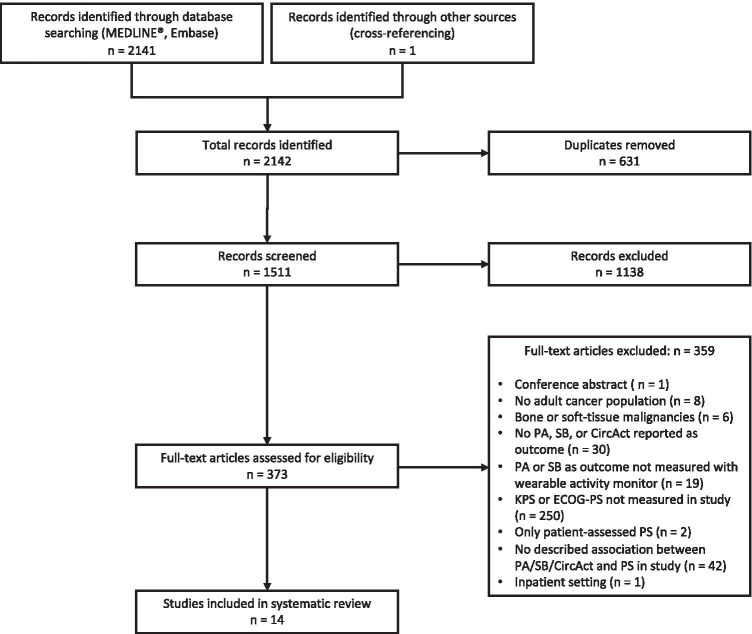
Flowchart of literature search and inclusion of studies. PA, physical activity; SB, sedentary behavior; KPS, Karnofsky Performance Status; ECOG-PS, Eastern Conference Oncology Group Performance Status; CircAct, circadian rest-activity rhythm; PS, performance status; *n*, number of studies

The combined literature search yielded 1511 unique records. The result of the systematic literature search and subsequent selection of studies is depicted in Fig. 1*.* After initial screening, 373 articles were retrieved in full-text and checked for eligibility. Cross-referencing provided one additional study [29] that could be included in the systematic review*.* Ultimately, 14 studies met the eligibility criteria and where included for further analysis. Table 1 provides an overview of the baseline characteristics of the included articles.

**Table 1:** Baseline characteristics of the included studies (*n* = 14)

Study first author (year)	n	Female	Age (years)	ECOG-PS	KPS	Cancer type	Disease stage	Treatment during study
Mormont (2000)	192	64 (33.3%)	58 [20–75]^a^	ECOG 0: 123 (64.1%) ECOG 1: 55 (28.6%) ECOG 2: 14 (7.3%)		Colorectal	Stage IV	Chemotherapy
Roscoe (2002)	78	78 (100%)	52 [34–79]^a^		89 [70–100]^a^	Breast		Chemotherapy
Innominato (2009)	130	56 (43.1%)	60 [22–76]^b^	ECOG 0: 70 (53.8%) ECOG 1: 45 (34.6%) ECOG 2: 15 (11.5%)		Colorectal	Stage IV	Chemotherapy
Ferriolli (2012)	53	13 (24.5%)	64 (9)^c^	ECOG 0: 19 (35.8%) ECOG 1: 22 (41.5%) ECOG 2: 11 (20.8%) ECOG 3: 1 (1.9%)	85 ± 11 [60–100]^d^	Upper gastrointestinal		Surgery: 37 (69.8%) Palliative chemotherapy: 16 (30.2%)
Maddocks (2012)	84	30 (35.7%)	66 [41–86]^a^	ECOG 0: 16 (19.0%) ECOG 1: 47 (56.0%) ECOG 2: 21 (25.0%)		NSCLC: 71 (84.5%) SCLC: 8 (9.5%) Mesothelioma: 5 (6.0%)	Stage IIIb: 43 (51.2%) Stage IV: 41 (48.8%)	No treatment past 4 weeks and scheduled for palliative treatment
Broderick (2014)	100	89 (89.0%)	54.7 [24–82]^a^	ECOG 0: 28 (28.0%) ECOG 1: 61 (61.0%) ECOG 2–3: 11 (11.0%)		Breast: 68 (68.0%) Lung/thoracic: 10 (10.0%) Gynecological: 10 (10.0%) Other: 12 (12.0%)		Chemotherapy
Lévi (2014) (cohort III)	142	55 (38.7%)	60 [21–83]^b^	ECOG 0: 84 (59.6%) ECOG 1: 44 (31.2%) ECOG 2: 13 (9.2%) Unknown: 1 (0.7%)		Colorectal	Stage IV	Chemotherapy
Jeffery (2017)	46	13 (28.3%)	68.5 (7.9)^c^	ECOG 0: 18 (39.1%) ECOG 1: 13 (28.3%) ECOG 2: 7 (15.2%) ECOG 3: 6 (13.0%)		Mesothelioma: 30 (65.2%) Lung cancer: 11 (23.9%) Other: 5 (10.9%)	Patients with malignant pleural effusion	Chemotherapy
Dennett (2018)	49	33 (67.3%)	63 [27–77]^a^		80.8 (10.4)^c^ 80 [60–100]^a^	Breast: 24 (49.0%) Prostate: 5 (10.2%) NHL: 5 (10.2%) Other: 16 (20.7%)*		No therapy: 19 (38.8%) Hormone therapy: 12 (24.5%) Chemotherapy: 20 (20.4%) Targeted therapy: 6 (12.2%) Radiotherapy: 5 (10.2%)**
Gresham (2018)	37	17 (45,9%)	62 [34–81]^a^	ECOG 0: 9 (24.3%) ECOG 1: 13 (35.1%) ECOG 2: 9 (24.3%) ECOG 3: 6 (16.2%)	KPS 100: 6 (16.2%) KPS 90: 5 (13.5%) KPS 80: 9 (24.3%) KPS 70: 8 (21.6%) KPS 60: 3 (8.1%) KPS 50: 5 (13.5%) KPS < 50: 1 (2.7%)	Pancreas: 27 (73.0%) Other gastrointestinal: 7 (18.9%) Other: 3 (8.1%)	Stage IIIB: 34 (91.9%) Stage IV: 3 (8.1%)	Any type of treatment
Gupta (2018)	24	16 (67.7%)	54 (12.5)^c^	ECOG 0: 13 (54.2%) ECOG 1: 9 (37.5%) ECOG 2: 2 (8.3%)		Gastrointestinal: 12 (50.0%) Breast: 4 (16.7%) Lung: 3 (12.5%) Other: 5 (20.8%)		Chemotherapy
Broderick (2019)	42	21 (50.0%)	48.2 [24–72]^a^	ECOG 0: 22 (52.4%) ECOG 1: 18 (42.9%) ECOG 2: 1 (2.4%) Unknown: 1 (2.4%)		Breast: 17 (40.5%) Testicular: 10 (23.8%) Head and neck: 7 (16.7%) Other: 8 (19.0%)	No evidence disease: 3 (7.1%) Locally recurrent: 14 (33.3%) Distant metastases: 21 (50%)	Chemotherapy
Fujisawa (2019)	41	21 (51.2%)	66.8 (10.6)^c^	ECOG 0: 9 (22.0%) ECOG 1: 26 (63.4%) ECOG 2: 5 (12.2%) ECOG 3: 1 (2.4%)		NSCLC	Stage IV	Any type of treatment
Ohri (2019)	50	20 (40.0%)	66 [38–90]^a^	ECOG 0: 11 (22.0%) ECOG 1: 33 (66.0%) ECOG 2: 6 (12.0%)		NSCLC	Stage II: 6 (12.0%) Stage IIIA: 24 (48.0%) Stage IIIB: 16 (32.0%) Stage IV: 4 (8.0%)	Chemoradiotherapy

^a^Mean [range]; ^b^Median [range]; ^c^Mean (SD); ^d^Mean ± SD [range]; *1 patient recorded 2 primary cancer types; **3 patients on combination of treatments

*ECOG-PS*, Eastern Cooperative Oncology Group; *PS*, performance status; *KPS*, Karnofsky Performance Status; *NSCLC*, non-small cell lung carcinoma; *SCLC*, small cell lung carcinoma; *NHL*, non-Hodgkin lymphoma

### Risk of bias assessment

Results of the risk of bias assessment are depicted in Table 2. The median methodological quality of the included studies was 40% and ranged from 20 to 60%. Only 29% of included studies had an adequate participation rate (> 80%). Most (64%) studies had a small sample size (*n* < 100). None of the studies adequately described methods used for dealing with missing physical activity data, and the majority of studies (79%) used a combination of device and wear-time protocol that has not been adequately validated in the studied population.

**Table 2:** Risk of bias assessment tool and quality score of the included studies

Items/reference		Topic	Mormont (2000)	Roscoe (2002)	Innominato (2009)	Ferriolli (2012)	Maddocks (2012)	Broderick (2014)	Lévi (2014)	Jeffery (2017)	Dennett (2018)	Gresham (2018)	Gupta (2018)	Broderick (2019)	Fujisawa (2019)	Ohri (2019)	Score (%)
*Study participation*																	
A	The sampling frame and recruitment are adequately described (e.g., setting and geographic location)	I	1	0	0	1	1	1	0	1	0	0	0	1	1	0	**50.0**
B	Inclusion and exclusion criteria are adequately described	I	0	0	1	0	1	1	1	0	1	1	1	1	1	1	**71.4**
C	The baseline study sample (participants) is adequately described for key characteristics (e.g., age, gender, tumor type, stage, treatment)	I	1	0	1	1	1	0	1	0	1	0	0	1	0	1	**57.1**
D	There is adequate participation in the study by eligible patients (> 80%) or differences between responders and non-responders is non-selective	V/P	0	0	1	0	0	0	1	0	0	0	0	0	1	1	**28.6**
*Study attrition*																	
E	Number of patients included in the analysis ≥ 100	V/P	1	0	1	0	0	1	1	0	0	0	0	0	0	0	**28.6**
F	Proportion of study sample completing the study and providing outcome data is adequate (> 80%) or differences between patients with and without outcome data is non-selective	V/P	1	0	1	0	1	1	1	1	1	1	1	0	1	1	**78.6**
*Wearable activity monitor metrics measurement*																	
G	The reported variables for physical activity and sedentary behavior are continuous or appropriate cut-points are used	V/P	1	1	1	1	1	0	1	0	0	1	1	1	0	1	**71.4**
H	The used device and wear-time protocol have established validity for reported wearable activity monitor metrics	V/P	0	0	0	1	0	0	0	0	1	0	0	1	0	0	**21.4**
I	Appropriate methods are used for dealing with missing data	V/P	0	0	0	0	0	0	0	0	0	0	0	0	0	0	**0.0**
*Performance status measurement*																	
J	Performance status is clearly defined and measured with a valid and reliable tool	V/P	1	1	1	1	1	1	1	1	1	1	1	1	1	1	**100.0**
*Confounding measurement and account*																	
K	Important potential confounders are measured and choice of confounders is adjusted for	I	0	0	1	0	0	0	1	0	1	1	0	0	0	0	**28.6**
L	Important potential confounders are accounted for in the analysis	V/P	0	0	0	0	0	0	0	0	1	0	0	0	0	0	**7.1**
*Analysis*																	
M	The statistical analysis is clearly described and appropriate	V/P	1	0	1	1	1	1	1	1	1	1	1	1	1	1	**92.9**
N	Outcomes are sufficiently presented (i.e., point estimates and measures of variability)	I	0	0	0	1	1	1	0	1	1	1	0	1	1	0	**57.1**
O	Appropriate multivariable analysis is used	V/P	0	0	0	0	0	0	0	0	1	0	0	0	0	0	**7.1**
**Total risk of bias score (%)**			**50**	**20**	**60**	**40**	**40**	**40**	**60**	**30**	**60**	**40**	**40**	**40**	**40**	**40**	

1, study provided information on the quality item and met the criterion; 0, study provided information on the quality item but did not meet the criterion; ?, study provided no or insufficient information on the quality item. *I*, informativeness; *V*, validity; *P*, precision

### Wearable activity monitors and physical activity/sedentary behavior metrics

The characteristics of the used wearable activity monitors and physical activity/sedentary behavior metrics are summarized in Table 3. A total of 9 different devices were used in the included articles and worn on the wrist (9 studies [29–37]), thigh (3 studies [38–40]), hip (1 study [41]), or waist (1 study [42]). Furthermore, different wear-time protocols were used between studies. Included articles reported on a total of 12 different wearable activity monitor metrics: 8 studies reported on steps taken [33–35, 37–41], 6 on sedentary behavior (i.e., posture-based and activity counts-based) [34, 36, 38, 39, 41, 42], 4 on mean daily activity counts [29–32], 3 on time spent in MVPA [40–42], 3 on the dichotomy in-bed versus out-of-bed index I < O [30–32], and 2 on time spent in light physical activity (LPA) [41, 42]. Other reported PA metrics included: time spent stepping [38–40], time spent standing [29, 31], distance walked [33], total metabolic expenditure per day [38], and daily floors climbed [33] (Table 3). Different methods, definitions, and cut-points were used for sedentary behavior, LPA, and MVPA based on the devices used.

**Table 3:** Characteristics of used wearable activity monitors and metrics in included studies

Study	WAM	Wear location	Wear-time protocol	WAM metrics	Cut-points for SB and PA intensities
Mormont (2000)	Mini-motionlogger actigraph	Wrist	Continuously for 72 h	Daily activity levels (cpm); I < O	
Roscoe (2002)	Mini-motionlogger actigraph	Wrist	Continuously for 72 h	Daily activity levels (cpm)	
Innominato (2009)	Mini-motionlogger actigraph	Wrist	Continuously for 72 h	Daily activity levels (cpm); I < O	
Ferriolli (2012)	activPAL accelerometer	Thigh	Continuously for 7 days. Only complete 24 h recording days were included in analysis	Daily steps, energy expenditure (METs/day), time spent sitting or lying/standing/stepping	
Maddocks (2012)	activPAL accelerometer	Thigh	Continuously for 8 days. Only complete 24 h recording days were included in analysis	Daily steps, time spent sitting or lying/standing/stepping per day	
Broderick (2014)	RT3 accelerometer	Waist	7 days during waking hours, with at least 3 valid wear days (≥ 12 h of available data)	Waking hours spent in SB/LPA/MVPA	No clear cut-point reported
Lévi (2014)	Mini-motionlogger actigraph	Wrist	Continuously for 72 h	Daily activity levels (cpm); I < O	
Jeffery (2017)	Actigraph GT3X +	Hip	Continuously for 7 days with at least 1 valid wear day (≥ 8 h of waking wear time)	Daily steps, time spent in SB/LPA/MVPA	Freedson cut-points [50, 51]
Dennett (2018)	activPAL accelerometer	Thigh	Continuously for 7 days with at least 6 full days of available data (24 h)	Daily steps, daily time spent in MVPA, daily time spent standing or stepping	MVPA: 100 steps per minute [52]
Gresham (2018)	Fitbit Charge HR	Wrist	Continuously for 7 day with at least 4 valid days of available data	Daily steps, daily distance walked, daily stairs climbed	
Gupta (2018)	Fitbit Flex	Wrist	Continuously during observation period (max 12 weeks)	Daily steps, sedentary time	No clear cut-point reported for sedentary time
Broderick (2019)	Microsoft band	Wrist	During waking hours for the duration of the study (60 days)	Steps per hour	
Fujisawa (2019)	Actiwatch 2	Wrist	Continuously for 4 days with at least 3 full days (24 h) of available data	Time spent awake immobile	Immobile: epoch with an activity score of zero while not asleep
Ohri (2019)	Garmin Vivofit	Wrist	Not reported	Daily steps	

*WAM*, wearable activity monitor; *SB*, sedentary behavior; *PA*, physical activity; *cpm*, counts per minute; *I* < *O*, dichotomous rest/activity parameter; *LPA*, light physical activity; *MVPA*, moderate-to-vigorous physical activity

### Physical activity and sedentary behavior outcomes per ECOG-PS group

Table 4 provides an overview of the included studies that reported on the physical activity and sedentary behavior outcomes per ECOG-PS group. All studies that reported on mean daily steps per ECOG-PS group found significant between-group differences with more daily steps in patients with better performance status. Moreover, two studies [38, 39] reported that patients with better performance status spent significantly less time sitting/lying (i.e., sedentary) and significantly more time standing and stepping as compared to patients with worse performance status. Three studies reporting on mean total activity counts per day and the circadian rest-activity dichotomous index I < O, all showed significantly more activity counts and per day and less circadian rhythm disruption in patients with better performance status [30–32]. Regarding intensity-based wearable activity monitor metrics, one study reported that patients with good performance status spent significantly more time in LPA and MVPA and significantly less time sedentary as compared to patients with poor performance status [41]. Conversely, another study did not show significant differences regarding time spent in LPA and MVPA between groups based on performance status [42]. This study, however, reported that patients with better performance status spent significantly less time sedentary as compared to patients with worse performance status.

**Table 4:** Physical activity and sedentary behavior outcomes from wearable activity monitors per ECOG-PS group

ECOG-PS	Steps per day^a^				*P*-value	Volume/intensity of PA, SB, or postures^b^						*P*-value
	0	1	2	3		0	1		2		3	
Mormont (2000)						MeanAct: n/r I < 0: n/r	MeanAct: n/r I < O: n/r		MeanAct: n/r I < O: n/r			0.04c < 0.001c
Innominato* (2009)						MeanAct: 110 [89–128] I < O: 98 [94–99]	MeanAct: 104 [78–122] I < O: 95 [91–99]		MeanAct: 94 [76–117] I < O: 95 [85–97]			0.047d 0.01d
Ferriolli* (2012)	6400 [5500–8600]	4400 [3100–6400]	2500 [1900–3300]		0 vs 1 = 0.009e 0 vs 2 < 0.001e 1 vs 2 = 0.009e	Sitting/lying: 18.9 h [18.2–19.9] Standing: 3.4 h [2.7–4.0] Stepping: 1.5 h [1.2–2.1]	Sitting/lying 19.9 h [19.0–20.6] Standing: 3.0 h [2.5–3.6] Stepping: 1.1 h [0.8–1.5]		Sitting/lying 21.3 h [21.2–22.4] Standing: 2.0 h [1.3–2.5] Stepping: 0.7 h [0.5–0.8]			Sitting/lying 1 vs 0 = 0.002e 2 vs 0 = 0.005e Standing 1 < 0 = 0.011e 2 < 0 = 0.005e Stepping 1 < 0 = 0.005**e** 2 < 0 = 0.001e 2 < 1 = 0.006e
Maddocks (2012)	8126 ± 3334	3791 ± 2064	2307 ± 1518		< 0.05f	Sitting/lying 17.7 h ± 2.2 Standing 4.3 h ± 1.9 Stepping 1.9 h ± 0.7	Sitting/lying 19.7 h ± 1.7 Standing 3.3 h ± 1.4 Stepping 1.0 h ± 0.5		Sitting/lying 21.0 h ± 1.7 Standing 2.4 h ± 1.4 Stepping 0.6 h ± 0.4			< 0.05f < 0.05f < 0.05f
Broderick (2014)						SB 7.5 h (6.5–8.5) LPA 4.7 h (4.0–5.5) MVPA 0.9 h (0.4–1.3)	SB 8.2 h (7.7–8.7) LPA 4.3 h (3.8–4.7) MVPA 0.6 h (0.4–0.8)		SB 9.2 h (8.4–10.0) LPA 3.7 h (2.5–5.0) MVPA 0.2 h (0.1–0.4)			0.04f 0.28f 0.09f
Lévi (2014)						MeanAct 110 ± 27 I < O 98.2 [95.4–99.3]	MeanAct 105 ± 29 I < O 96.5 [93.1–99.0]		MeanAct 84 ± 35		MeanAct 42 ± 44	< 0.05c < 0.001c
									I < O 91.5 [79.1–97]			
Jeffery (2017)	6851 ± 2948	6317 ± 2786	2866 ± 2881	3334 ± 1405	0–1 vs 2–3 < 0.001**g**	SED 68.8% ± 10.2 LPA 30.1% ± 10.8 MVPA 0.9% [0.32–1.43]			SED 80.1% ± 6.32 LPA 19.7% ± 6.24 MVPA 0.1% [0.05–0.25]			0–1 vs 2–3 = 0.010** g** 0–1 vs 2–3 = 0.003** g** 0–1 vs 2–3 < 0.001** g**
Gresham* (2018)	4600 [3600–7800]	4800 [1900–6600]	1600 [900–2000]	1100 [400–1300]	< 0.0001							
Gupta (2018)	5911 ± 3358	1890 ± 1138	845 ± 555		0.002 0 vs 1 = 0.002 0 vs 2 = 0.044 1 vs 2 = 0.237							
Fujisawa (2019)						SB 18.0 h ± 10.6						
Ohri (2019)	7788 [6578–12075]	5221 [3531–7845]	1066 [759–4146]		< 0.01c							

^*^Data extracted from graphs using WebPlotDigitizer. Data on steps per day obtained this way are rounded to the nearest hundred. ^a^Values represent mean ± SD or median [IQR]. ^b^Values represent mean (95%CI), mean ± SD, or median [IQR]. ^c^Kruskal-Wallis test. ^d^Jonckheere’s trend test. ^e^Independent Student’s *t*-test. ^f^ANOVA test. ^g^Mann-Whitney *U* test. *PA*, physical activity; *SB*, sedentary behavior; *ECOG*, Eastern Cooperative Oncology Group; *PS*, performance status; *n/r*, not reported; *MeanAct*, meant daily activity level; *I* < *O*, dichotomous circadian disruption parameter; *SB*, sedentary behavior; *LPA*, light physical activity; *MVPA*, moderate-to-vigorous physical activity

### Evidence synthesis for associations between wearable activity monitor metrics and performance status

In total, we found 14 studies that could be included in the evidence synthesis. Results of evidence synthesis for the association between wearable activity monitor metrics and performance status are compiled in Table 5. We found moderate evidence for a moderate positive association between daily steps and performance status and moderate evidence for a weak positive association between activity counts and performance status. Moreover, we found moderate evidence for moderate positive associations between time spent standing/stepping and performance status and between the circadian rest-activity dichotomous index I < O and performance status. Finally, we found moderate evidence for moderate negative associations between sedentary behavior (intensity- or posture-based) and performance status.

**Table 5:**
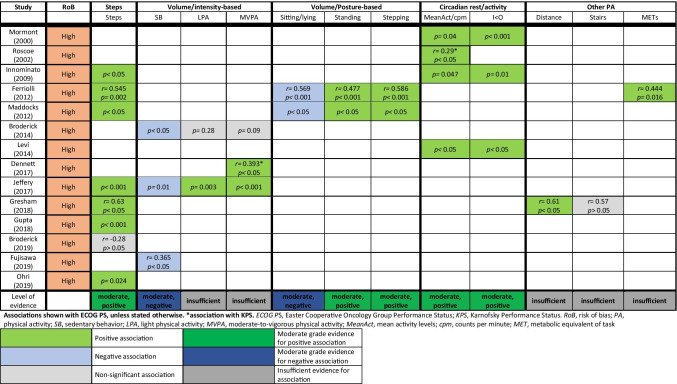
Evidence synthesis for association between wearable activity monitor metrics and performance status

**Associations shown with ECOG PS, unless stated otherwise.** *association with KPS. *ECOG PS*, Easter Cooperative Oncology Group Performance Status; *KPS*, Karnofsky Performance Status. *RoB*, Risk of bias; *PA*, physical activity; *SB*, sedentary behavior; *LPA*, light physical activity; *MVPA*, moderate-to-vigorous physical activity; *MeanAct*, mean activity levels; *cpm*, counts per minute; *MET*, metabolic equivalent of task

## Discussion

In this study, we reviewed the available evidence on the association between wearable activity monitor metrics and physician-assessed performance status. Evidence synthesis showed moderate evidence for weak-to-moderate positive associations between performance status and various wearable activity monitor metrics and a moderate negative association between performance status and sedentary behavior.

Different possible explanations can be provided for the absence of strong associations. First, these weak-to-moderate associations may suggest that wearable activity monitors and performance status scales assess different constructs of physical performance and cannot simply be interchanged. Wearable activity monitors objectively measure physical activity (levels) and can therefore be regarded as performance-based measurements that are independent of judgment. Performance status scales, on the other hand, are evaluation-based measurements that involve judgment using idiosyncratic criteria [5, 6]. Another possible explanation for the absence of strong associations can also be provided from a measurement perspective, with only few categories for physicians-assessed performance status. In the studies included in this review, only 12% of patients had poor performance status (ECOG-PS 2–3). The limited variation in scoring could have contributed to the absent, or weak-to-moderate associations. Furthermore, substantial heterogeneity across studies in terms of devices used, wear-time protocols, study population, and methodology could be a potential source for the absence of strong associations between wearable activity monitor metrics and performance status.

More detailed objectively and passively gathered activity data from wearable activity monitors might be of added value in clinical practice. Wearable activity monitor-assessed physical activity/sedentary behavior might serve as a dynamic and objective supplement measurement of patients’ performance status as assessed by the physician and, as such, might prove to be of added value in clinical decision making and evaluation of treatment options in oncology. This hypothesis is substantiated by observations that more daily steps are associated with lower risk of hospitalization during cancer treatments [33, 43], longer survival [33, 41, 44], and lower chance of serious adverse events [33, 45]. Interestingly, Fujisawa et al. demonstrated that among patients with good performance status (ECOG-PS 0–1), ECOG-PS was not predictive for survival, while sedentary behavior was a significant predictor for 6-month survival [36]. Moreover, Jeffery et al. reported that patients with a survival longer than 3 months spent significantly less time sedentary as compared to those who survived less than 3 months [41]. Together, these results suggest potential value of objective sedentary behavior measurement in predicting survival outcomes, especially in patients with good PS. In this way, wearable activity monitors might assist physicians in clinical decision making, like determining whether a patient is fit for treatment.

Most currently available wearable activity monitors (e.g., Fitbit Charge HR) are multisensory devices that have a built-in 3D-accelerometer as well as other sensors that measure, for example, heart rate. In the era of advancing artificial intelligence and machine learning, it is very conceivable that data input from combinations of wearable activity monitor sensors and data patterns over time might prove to be superior in assessing performance status and predicting outcomes for patients with cancer than physician-assessed performance status.

Recently, various pilot studies have demonstrated the feasibility of using wearable activity monitors in the context of an ambulatory monitoring platform that longitudinally assesses treatment-related adverse events, unplanned healthcare encounters, and survival in patients with cancer [33, 46, 47]. Results from these studies suggest potential for wearable activity monitors in early detection of adverse event and unplanned healthcare encounters. The application of wearable activity monitors in this context has a lot of potential to be clinically impactful and improve cancer care. Future research should focus on proving the efficacy of wearable activity monitors as a part of ambulatory monitoring platforms.

An important finding of our systematic review is the high heterogeneity between included studies regarding study population, devices used, wear-time protocols, definitions and cut-points used for different physical activity metrics, and reporting of outcomes, thereby hindering adequate comparison of results on the association between physical activity metrics and performance status and complicating best evidence synthesis. A second limitation of the studies included in this review is the high risk of bias scores. Major factors contributing to the high risk of bias scores were unvalidated methods regarding physical activity measurements, low sample sizes, and the lack of multivariable analyses to adjust for relevant confounders. It should be noted that the majority of these studies investigated the association between wearable activity monitor metrics and performance status as secondary or exploratory analysis, which may have contributed to the high risk of bias scores. Consequently, it is currently also unclear whether the association between wearable activity monitor metrics and performance status varies by cancer type or stage. With regard to the physical activity measurements, none of the studies adequately reported on the handling of missing physical activity data. Different studies have emphasized the need for missing accelerometer data imputation and suggested statistical methods of handling missing data [48, 49]. Moreover, the majority of studies used devices, wear-time protocols, or cutoff points that have not adequately been validated in comparable populations. Taken together, results should be interpreted with caution and emphasize that standardization of wearable activity monitor-measured physical activity and sedentary behavior methodology is warranted to decrease risk of bias in future studies on the subject.

Strengths of this systematic review include the in-depth risk of bias assessment that was adjusted specifically for studies using a wearable activity monitor for physical activity and sedentary behavior measurements and the subsequent best evidence synthesis. However, most of the included studies were not designed to investigate the association between wearable activity monitor metrics and performance status, complicating risk of bias assessment and evidence synthesis. More than half of the included studies were designed to investigate physical activity levels in specific cancer populations, study the feasibility of wearable activity monitors, or explore associations between other wearable activity monitor metrics, like circadian rest-activity rhythm parameters, and various outcomes. Therefore, the association between wearable activity monitor metrics and performance status was often analyzed in a secondary or exploratory analysis resulting in suboptimal presenting of results. Moreover, results may be prone to reporting bias as non-significant associations are less likely to be reported, resulting in an overestimation of the associations between wearable activity monitor metrics and performance status.

In conclusion, we found moderate evidence for a positive weak-to-moderate association between various physical activity metrics and performance status and for an inverse moderate association between sedentary behavior and performance status. The strength of the associations should be interpreted with caution given the aforementioned limitations of the available evidence. Nevertheless, our results suggest that objectively measured physical activity may serve as a dynamic and objective supplement measurement of a patient’s functional performance status and may be of added value in clinical decision making and evaluation of treatment options in oncology. Next steps include to study the association between wearable activity monitor metrics and clinical outcomes and directly compare the predictive value of objectively measured physical activity with performance status for relevant clinical outcomes. Finally, consensus is warranted on the methodology of objective physical activity measurement and efforts should be made to validate the different methods (i.e., device, parameters, wear-time protocols) in relevant patient populations.

## Supplementary Information

Below is the link to the electronic supplementary material.

Supplementary file 1 (PDF 91 KB)

## Data Availability

Data are available on request from the authors.
