# Supplementary material for: The association between wearable activity monitor metrics and performance status in oncology: a systematic review
Source: Support Care Cancer. 2021 Jun 12;29(11):7085–99. doi: 10.1007/s00520-021-06234-5 (PMC8464563; doi:10.1007/s00520-021-06234-5)
Supplement: Supplementary file 1 — (PDF 91 KB) [file 520_2021_6234_MOESM1_ESM.pdf]

Supplementary Table 1. Search strategy

	Medline via Pubmed		EMBASE via Ovid
#1	"Wearable electronic device"[MESH] OR wearable electronic device*[tiab] OR wearable device*[tiab] OR wearable technolog*[tiab] OR wearable sensor*[tiab] OR wearable biosensor*[tiab] OR wearable monitor*[tiab] OR wireless sensor*[tiab] OR wireless biosensor*[tiab] OR wireless electronic device*[tiab] OR wireless device*[tiab] OR wireless technolog*[tiab] OR wireless monitor*[tiab] OR electronic skin[tiab] OR activity tracker*[tiab] OR activity monitor*[tiab] OR fitness tracker*[tiab] OR fitness monitor*[tiab] OR smartwatch*[tiab] OR fitbit*[tiab] OR apple watch*[tiab] OR Jawbone*[tiab] OR Pebble*[tiab] OR pedometer*[tiab] OR accelerometer*[tiab] OR Actigraph*[tiab] OR remote monitor*[tiab] OR ambulatory monitor*[tiab] OR outpatient monitor*[tiab] OR actiwatch*[tiab] OR patch sensor*[tiab] OR skin sensor*[tiab] OR skin biosensor*[tiab] OR smartphone*[tiab] OR Iphone[tiab] OR Android[tiab]	1.	exp accelerometer/ or exp pedometer/ or exp activity tracker/ or exp smartphone/ or exp electronic device/ or (wearable electronic device* or wearable device* or wearable technolog* or wearable sensor* or wearable biosensor* or wearable monitor* or wireless sensor* or wireless biosensor* or wireless electronic device* or wireless device* or wireless technolog* or wireless monitor* or electronic skin or activity tracker* or activity monitor* or fitness tracker* or fitness monitor* or smartwatch* or fitbit* or apple watch* or Jawbone* or Pebble* or pedometer* or accelerometer* or Actigraph* or remote monitor* or ambulatory monitor* or outpatient monitor* or actiwatch* or patch sensor* or skin sensor* or skin biosensor* or smartphone* or Iphone or Android).ti,ab,kw.
#2	"Neoplasms"[Mesh] OR cancer[sb] OR oncolog*[tiab] OR cancer*[tiab] OR neoplas*[tiab] OR tumour*[tiab] OR tumor[tiab] OR tumors[tiab] OR carcinom*[tiab] OR melanom*[tiab] OR lymphom*[tiab] OR leukemi*[tiab] OR malignan*[tiab] OR metasta*[tiab] OR carcinogen*[tiab] OR oncogen*[tiab] OR anticarcinogen*[tiab] OR sarcoma*[tiab] OR precancerous[tiab] OR paraneoplastic[tiab] OR neuroma*[tiab] OR blastoma*[tiab] OR meningioma*[tiab] OR lymphangioma*[tiab] OR lymphangiomyoma*[tiab] OR lymphangiosarcoma*[tiab] OR hodgkin disease*[tiab] OR plasmacytoma*[tiab] OR carcinosarcoma*[tiab] OR hepatoblastoma*[tiab] OR mesenchymoma*[tiab] OR chordoma*[tiab] OR germinoma*[tiab] OR gonadoblastoma*[tiab] OR mesonephroma*[tiab] OR teratoma*[tiab] OR teratocarcinoma*[tiab]	2.	exp oncology/ or exp neoplasm/ or (oncolog* or cancer* or neoplas* or tumour* or tumor or tumors or carcinom* or melanom* or lymphom* or leukemi* or malignan* or metasta* or carcinogen* or oncogen* or anticarcinogen* or sarcoma* or precancerous or paraneoplastic or neuroma* or blastoma* or meningioma* or lymphangioma* or lymphangiomyoma* or lymphangiosarcoma* or hodgkin disease* or plasmacytoma* or carcinosarcoma* or hepatoblastoma* or mesenchymoma* or chordoma* or germinoma* or gonadoblastoma* or mesonephroma* or teratoma* or teratocarcinoma*).ti,ab,kw.
#3	"Exercise"[Mesh] OR "Walking"[Mesh] OR Physical activit*[tiab] OR activity monitor*[tiab] OR daily activit*[tiab] OR activity data[tiab] OR activity metric*[tiab] OR exercis*[tiab] OR step count*[tiab] OR counted step*[tiab] OR steps taken[tiab] OR taken steps[tiab] OR taken step[tiab] OR steps per[tiab] OR step count per[tiab] OR accelerometer*[tiab] OR pedometer*[tiab] OR MVPA[tiab] OR sedentar*[tiab] OR immobility*[tiab] OR motor activit*[tiab] OR daily steps*[tiab] OR weekly steps*[tiab] OR daily step count[tiab] OR weekly step count[tiab] OR walk*[tiab]	3.	exp physical activity/ or (Physical activit* or activity monitor* or daily activit* or activity data or activity metric* or exercis* or step count* or counted step* or steps taken or taken steps or taken step or steps per or step count per or accelerometer* or pedometer* or MVPA or sedentar* or immobility* or motor activit* or daily steps* or weekly steps* or daily step count or weekly step count or walk*).ti,ab,kw.
#4	#1 AND #2 AND #3	4.	1 and 2 and 3
#5	(#4) AND English [Language]	5.	limit 4 to conference abstract status
		6.	4 not 5
		7.	limit 6 to (english language)

Supplementary Table 2. Risk of bias assessment list and description

Item	Description	Explanation	Topic
Study participation			
A	The sampling frame and recruitment are adequately described	Clear description of recruitment period, setting, and geographic location	I
B	Inclusion and exclusion criteria are adequately described	clear definitions provided for inclusion and exclusion criteria	I
C	The baseline study sample (participants) are adequately described for key characteristics	At least: age, gender, tumor type, stage, treatment, comorbidities	I
D	There is adequate participation in the study by eligible patients (>80%) or differences between responders and non-responders is non-selective	attrition analyses were performed and showed no significant differences between baseline study sample and population of eligible subject	V/P
Study attrition			
E	Number of patients included in the analysis ≥ 100	self-explanatory	V/P
F	Proportion of study sample completing the study and providing outcome data is adequate (> 80%) or differences between patients with and without outcome data is non-selective	attrition analyses were performed and showed no significant differences between patients with and without outcome data	V/P
Physical activity measurement			
G	The reported variables for physical activity and sedentary behavior are continuous or appropriate cut-points are used	cut-points are clearly defined, not data-dependent, and have established validity in comparable populations	V/P
H	The used device and wear-time protocol have established validity for reported physical activity variables	Validation studies have been published for the used devices and wear-time protocols in comparable populations	V/P
I	Appropriate methods are used for dealing with missing physical activity data	Adequate imputation techniques were used to replace missing physical activity data	V/P
Performance status measurement			
J	Performance status is clearly defined and measured with a valid and reliable tool	healthcare professional-assessed ECOG-PS or KPS score	V/P
Confounding measurement and account			
K	Important potential confounders are measured and choice of confounders is adjusted for	self-explanatory	I
L	Important potential confounders are accounted for in the analysis	Results are adjusted for sedentary behavior and/or physical activity	V/P
Analysis			
M	The statistical analysis is clearly described and appropriate	self-explanatory	V/P
N	Outcomes are sufficiently presented (i.e. point estimates and measures of variability)	self-explanatory	I
O	Appropriate multivariable analysis is used	The number of samples is at least 10 times the number of independent variables	V/P

I, Informativeness; V, validity; P, precision.
